# Factors and outcomes associated with discontinuation of basal insulin therapy in patients with type 2 diabetes mellitus

**DOI:** 10.1002/edm2.122

**Published:** 2020-03-12

**Authors:** Puhong Zhang, Heng Zhang, Xian Li, Minyuan Chen, Du Wang, Linong Ji

**Affiliations:** ^1^ The George Institute for Global Health at Peking University Health Science Center Beijing China; ^2^ Department of Endocrinology and Metabolism Peking University People's Hospital Beijing China

**Keywords:** basal insulin, insulin discontinuation, type 2 diabetes mellitus

## Abstract

**Objectives:**

To assess treatment discontinuation, associated factors and outcomes after initiating basal insulin (BI) among Chinese insulin‐naïve patients with type 2 diabetes mellitus (T2DM) who had previously uncontrolled hyperglycaemia on OADs.

**Patients:**

Insulin‐naïve patients with T2DM who had uncontrolled hyperglycaemia (HbA1c ≥7%) by OADs and were willing to initiate BI treatment were enrolled from 209 secondary and tertiary hospitals in eight geographical regions in China.

**Design:**

Each participant was interviewed at baseline, 3 and 6 months to collect study information. Patients with at least one visit during follow‐up were included in the analyses. BI discontinuation was defined by a question asking whether the patient discontinued BI therapy at 3 or 6 months. Analyses were conducted to identify baseline factors associated with BI discontinuation and to estimate the association between insulin treatment discontinuation and patients' clinical outcomes at 6 months.

**Results:**

Of 17 858 patients, 25.8% discontinued basal insulin therapy within 6 months after initiation, and nearly two‐thirds doing so within the first 3 months. Among patients discontinued basal insulin, 70.2% stopped all insulin therapy; 25.9% switched to premixed insulin and 3.8% switched to bolus only. Three most common reasons for BI discontinuation reported by patients were being unwilling to persist basal insulin without specific reasons (46.8%), reducing the frequency of daily injection (23.5%) and medical affordability (15.1%). Factors significantly associated with BI discontinuation were hospital level, patient recruitment setting, age, education level, out‐of‐pocket ratio, BMI, diabetes duration, self‐monitoring of blood glucose (SMBG), numbers of OADs, BI type and insulin regimen. Compared with discontinuers, patients continued BI therapy had higher FPG (46.4% vs 28.8%) and HbA1c (42.3% vs 36.5%) control rate.

**Conclusion:**

Among patients with T2DM who initiated BI therapy due to uncontrolled hyperglycaemia by OADs, the proportion of insulin discontinuation was high within 6 months. Further study is needed to understand the reason behind the BI discontinuation.

## Graphical abstract

In this study, a quarter of patients with type 2 diabetes mellitus who initiated basal insulin (BI) therapy due to uncontrolled hyperglycaemia by OADs discontinued BI therapy within 6‐month follow‐up. The most common reason for discontinuation reported by patients was unwilling to persist basal insulin without specific reasons.

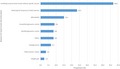

## 1 INTRODUCTION

Type 2 diabetes mellitus (T2DM) is the most common form of diabetes, which is characterized by the progressive deterioration in pancreatic beta cell function, leading to hyperglycaemia in the context of insulin resistance and/or insulin deficiency.^1^ Strong evidence from ADVANCE and UKPDS studies both showed that maintaining strict glycaemic control can significantly reduce the risk of microvascular disease.^2^, ^3^ Ten‐year follow‐up UKPDS also indicated that the improved glycaemic control can prevent myocardial infarction and any causes of death.^4^ Stepwise treatment intensification to maintain glycaemic control was recommended by the treatment guideline for patients with T2DM, which began with lifestyle changes, followed by the addition of metformin and other oral antidiabetic drugs (OADs). Insulin will be eventually required in most cases due to the progressive nature of the disease.^5^

However, a substantial proportion of patients do not achieve appropriate glycaemic control after initiating insulin treatment.^6^, ^7^ This may partly be attributed to poor persistence to insulin therapy,^8^, ^9^ defined as the duration of time from initiation to discontinuation of treatment or the proportion of patients who continued treatment for a specific time.^10^ Previous studies also revealed discontinued basal insulin (BI) treatment incurred higher medical resource use and costs than continuers.^11^, ^12^, ^13^ While several studies have examined factors associated with insulin treatment persistence such as age, comorbidity, type of initiated insulin and concomitant antidiabetic drug use,^8^, ^13^, ^14^ few studies have explored the patients' reasons for discontinuing treatment. In addition, most studies assessed persistence patterns using retrospective claims rather than actual medication‐taking behaviour.^8^, ^11^, ^12^, ^15^ The limited information hinders better management of treatment after initiating insulin to improve patients' persistence with insulin treatment.

The objective of this study was to assess treatment persistence, associated factors and outcomes after initiating BI among Chinese insulin‐naïve patients with T2DM who had previously uncontrolled hyperglycaemia on OADs, based on a prospective real‐world study, the Observational Registry for Basal Insulin Treatment study (ORBIT study).

## 2 METHODS

### 2.1 Study design and patients

This study was a subanalysis of the Observational Registry for Basal Insulin Treatment study (ORBIT study),^16^ which was a prospective, observational registry study focusing on the real‐world use, effectiveness and safety of initial BI regimen in type 2 diabetic patients uncontrolled by oral antidiabetic drugs in China. From December 2011 to June 2013, 18 995 eligible insulin‐naïve patients with T2DM who had uncontrolled glycaemia (HbA1c ≥7%) by OADs and willing to initiate BI treatment were enrolled by their providers from 209 secondary and tertiary hospitals in eight geographical regions in China. Each participant was interviewed at baseline (visit 1, v1), 3 months (visit 2, v2) and 6 months (visit 3, v3) to collect study information. Patients with at least one visit during follow‐up were included in the analyses.

Eligible patients were 18‐80 years with type 2 diabetes who had uncontrolled hyperglycaemia by OADs (HbA1c ≥7% measured within 3 months), and therefore, they initiated BI (glargine, detemir or NPH) treatment as their first insulin, at the physicians' discretion and their willingness.

Individuals were excluded if they met one of the following criteria: (a) used any type of insulin in the past 2 years (except for the intermittent use shorter than 1 month each time); (b) clinically significant acute major organ or systemic disease or other condition judged by the investigator that would create difficulty for the 6‐month follow‐up; (c) current or planned pregnancy or lactating women; and (d) involved in another clinical trial at least 1 month before the study enrolment.

The ORBIT study protocol was approved by the Institutional Review Board (IRB) of Peking University and, when necessary, by local IRBs. Written informed consents were obtained from all patients.

### 2.2 Baseline measures

Data from each participant were collected through study‐specific records and traditional medical forms, consisting of demographic characteristics (age, gender, education level and location), clinical characteristics (BMI, diabetes diagnose date, OADs initiation date, type of OADs used before, diabetes complications, self‐monitoring of blood glucose (SMBG) and hypoglycaemia events) and current treatment regimen (type of OADs and insulin). Baseline fasting plasma glucose (FPG) and HbA1c were tested, and physical examinations (body weight and height) were recorded at each hospital before initiating BI.

### 2.3 End‐point measures

Discontinuation of BI was defined by a question asking whether the patient continued BI therapy, which was answered by physicians at v2 and v3, respectively. Patients who continued BI therapy at the time of interview were defined as persistence of BI, whereas those who had stopped insulin therapy or switched to other insulin without BI such as premixed were defined as discontinuation of BI. For those discontinuers, physicians recorded the date of discontinuation and asked patients to choose up to three reasons for the discontinuation, which were affordability, unsatisfied blood glucose control, hypoglycaemia, weight gain, unwilling to persist insulin therapy without specific reasons, reducing the frequency of injection and other reasons (specific description required). After analysing the description of other reasons, satisfied glycaemic control and following doctor's advice were screened as the additional two reasons of BI treatment discontinuation. FPG and HbA1c were tested, and physical examinations (body weight and height) were recorded at each hospital during 6 months.

For discontinuers, treatment persistent days were defined as the days between baseline and the date of discontinuation. For continuers, treatment persistent days were defined as the days between baseline and the date of last visit.

### 2.4 Statistical analyses

Descriptive statistics were analysed as frequencies (*n*) and percentages (%) for categorical variables; means and SD (median and interquartile range for skewed distribution) for continuous variables. Student's *t* tests or chi‐square tests were used to assess differences in baseline characteristics between BI continuers and discontinuers, as appropriate. Student's *t* tests and multi‐way ANOVA fitted with regions, hospital level (secondary or tertiary), patient recruitment setting (inpatient or outpatient clinic), age, education level, location (rural or urban), BMI at v3, duration of diabetes, SMBG frequency at v3 and number of OAD types at v3 were used to assess differences in end‐point outcomes between BI continuers and discontinuers. Kaplan‐Meier curves and log‐rank tests were used to analyse BI persistence by BI type and insulin regiment at treatment initiation. Univariate und multivariate Cox regression models were fitted to identify significant factors associated with time to treatment discontinuation. Stepwise regression models were fitted with regions as fixed variables (forced entry), and other variables included were hospital level (secondary or tertiary), patient recruitment setting (inpatient or outpatient clinic), age, gender, education level, location (rural or urban), BMI, duration of diabetes, number of complications, baseline HbA1c, SMBG frequency, general hypoglycaemia, number of OAD types before initiating BI and prescribed at visit 1, type of BI, insulin regimen and out‐of‐pocket ratio. Selection and deletion thresholds of variables in multivariate model were *P* < .10 and *P* < .15, respectively. Statistical significance was set at *P* < .05 using two‐sided tests. All statistical analyses were performed using sas version 9.4 (SAS Institute).

## 3 RESULTS

### 3.1 Demographics and baseline clinical characteristics

A total of 17 858 eligible patients were included, of which 50.3% and 49.7% were recruited from secondary and tertiary hospitals, respectively, and more than half (55.6%) were inpatients. Males accounted for 52.6% and the mean patient age was 55.4 years (Table 1).

**Table 1:** Baseline characteristics

Baseline variables	Total (N = 17 858) (%)	Basal insulin discontinuation (N = 4616) (%)	Basal insulin continuation (N = 13 242) (%)	*P*‐value
Overall	100.0	4616 (25.8)	13 242 (74.2)	
Region				
Northeast	1556 (8.7)	433 (9.4)	1123 (8.5)	<.0001
North coast	3124 (17.5)	836 (18.1)	2288 (17.3)	
Yellow River	2743 (15.4)	640 (13.9)	2103 (15.9)	
South coast	1844 (10.3)	660 (14.3)	1184 (8.9)	
Southwest	2546 (14.3)	773 (16.8)	1773 (13.4)	
East coast	2179 (12.2)	562 (12.2)	1617 (12.2)	
Yangtze River	2854 (16.0)	597 (12.9)	2257 (17.0)	
Northwest	1012 (5.7)	115 (2.5)	897 (6.8)	
Level of hospital initiating BI				
Secondary hospital	8975 (50.3)	2211 (47.9)	6764 (51.1)	.0002
Tertiary hospital	8883 (49.7)	2405 (52.1)	6478 (48.9)	
Patient recruitment settings				
Outpatient clinic	7923 (44.4)	1693 (36.7)	6230 (47.1)	<.0001
Inpatient ward	9935 (55.6)	2923 (63.3)	7012 (52.9)	
Age (y), mean ± SD	55.4 ± 10.3	54.9 ± 10.4	55.6 ± 10.3	.0001
Male	52.6	50.4	53.4	.0005
Education				
Primary school or illiterate	4791 (26.8)	1465 (31.7)	3326 (25.1)	<.0001
Junior high school	5495 (30.8)	1433 (31.0)	4062 (30.7)	
Senior high school	4489 (25.1)	1067 (23.1)	3422 (25.8)	
Junior college or higher	3083 (17.3)	651 (14.1)	2432 (18.4)	
Location				
Urban	12 222 (68.4)	2984 (64.6)	9238 (69.8)	<.0001
Rural	5636 (31.6)	1632 (35.4)	4004 (30.2)	
Out‐of‐pocket ratio (%), mean ± SD	41.6 ± 27.1	44.17 ± 28.1	40.7 ± 26.7	<.0001
BMI group (kg/m^2^), mean ± SD	24.7 ± 3.4	24.7 ± 3.3	24.8 ± 3.5	.0569
<24	7639 (42.8)	1973 (42.7)	5666 (42.8)	.0065
24‐27	7474 (41.9)	1871 (40.5)	5603 (42.3)	
≥28	2744 (15.4)	772 (16.7)	1972 (14.9)	
Diabetes duration (y), mean ± SD	6.4 ± 5.3	5.8 ± 5.2	6.7 ± 5.3	<.0001
Complications in the past (yes) (%)	36.3	36.0	36.4	.5938
Times of self‐monitoring of blood glucose (past month), median (IQR)	2.0 (5.0)	2.0 (4.0)	2.0 (6.0)	<.0001
0	6057 (33.9)	1751 (37.9)	4306 (32.5)	<.0001
1‐5	7468 (41.8)	1909 (41.4)	5559 (42.0)	
≥6	4333 (24.3)	956 (20.7)	3377 (25.5)	
HbA1c (%)	9.6 ± 2.0	9.9 ± 2.0	9.5 ± 2.0	<.0001
FPG (mmol/L)	11.6 ± 4.0	12.1 ± 4.3	11.5 ± 3.8	<.0001
General hypoglycaemia (past month)	990 (5.5)	267 (5.8)	723 (5.5)	.4070
OAD type before initiating BI (%)				
Metformin	11 683 (65.4)	3112 (67.4)	8571 (64.7)	.0009
Sulphonylureas	8150 (45.6)	2063 (44.7)	6087 (46.0)	.1342
α‐glycosidase inhibitors	4316 (24.2)	1028 (22.3)	3288 (24.8)	0.0005
Glinides	2569 (14.4)	564 (12.2)	2005 (15.1)	<.0001
Thiazolidinediones	991 (5.5)	232 (5.0)	759 (5.7)	.0713
Others	1725 (9.7)	483 (10.5)	1242 (9.4)	.0317
Numbers of OAD before initiating BI				
1 OAD	8178 (45.8)	2225 (48.2)	5953 (45.0)	.0007
2 OADs	7895 (44.2)	1946 (42.2)	5949 (44.9)	
≥3 OADs	1785 (10.0)	445 (9.6)	1340 (10.1)	
Types of OADs prescribed at v1 (%)				
Metformin	8453 (47.3)	2067 (44.8)	6386 (48.2)	<.0001
Sulphonylureas	4036 (22.6)	805 (17.4)	3231 (24.4)	<.0001
α‐glycosidase inhibitors	5292 (29.6)	1236 (26.8)	4056 (30.6)	<.0001
Glinides	2569 (14.4)	564 (12.2)	2005 (15.1)	<.0001
Thiazolidinediones	991 (5.5)	232 (5.0)	759 (5.7)	.0713
Others	1725 (9.7)	483 (10.5)	1242 (9.4)	.0317
Numbers of OAD prescribed at v1				
None	3577 (20.0)	1311 (28.4)	2266 (17.1)	<.0001
1 OAD	7668 (42.9)	1805 (39.1)	5863 (44.3)	
2 OADs	6613 (37.0)	1500 (32.5)	5113 (38.6)	
BI type				
Glargine	12 402 (69.4)	2936 (63.6)	9466 (71.5)	<.0001
Detemir	2282 (12.8)	522 (11.3)	1760 (13.3)	
NPH	3174 (17.8)	1158 (25.1)	2016 (15.2)	
Dose of basal insulin (IU/kg/d), mean ± SD	0.17 ± 0.07	0.17 ± 0.07	0.18 ± 0.07	<.0001
Total insulin dose (IU/kg/d), mean ± SD	0.27 ± 0.20	0.31 ± 0.22	0.25 ± 0.18	<.0001
Prandial insulin at baseline	4366 (24.4)	1796 (38.9)	2570 (19.4)	<.0001
Injection numbers of prandial per day at baseline				
1 injection	32 (0.7)	16 (0.6)	16 (0.9)	.2956
2 injections	60 (1.4)	26 (1.3)	34 (1.5)	
3 injections	4274 (97.9)	1754 (98.0)	2520 (97.7)	

Patients had a mean diabetes duration of 6.4 years and 36.3% of them had complications in the past. The median SMBG frequency was 2 times per month. The mean HbA1c and FPG levels were 9.6% and 11.6 mmol/L, respectively. Before initiating BI treatment, metformin was the most commonly used OAD, which accounted for 65.4%, followed by sulphonylureas (45.6%) and α‐glycosidase inhibitors (24.2%). After initiating BI treatment, metformin accounted for 47.3%, and the proportion of α‐glycosidase (29.6%) was a bit higher than sulphonylureas (22.6%). Glargine, detemir and NPH accounted for 69.4%, 12.8% and 17.8%, respectively, as the three types for BI. Nearly a quarter of patients initiated basal‐bolus insulin treatment, in which 97.9% (4274/4366) used a full basal‐bolus regimen (three injections of bolus insulin per day) (Table 1).

### 3.2 BI treatment persistence and associated end‐point characteristics

Table 2 shows that 3016 (16.9%) and 4616 (25.8%) patients discontinued BI treatment at v2 and v3, respectively. In those who discontinued BI at v2, 112 (3.7%) and 881 (29.2%) switched to bolus only and premixed, respectively, whereas 2023 (67.1%) stopped all insulin therapy. And at v3, 177 (3.8%) and 1197 (25.9%) switched to bolus only and premixed, respectively, whereas 3242 (70.2%) stopped all insulin therapy.

**Table 2:** Insulin regimen after discontinuation of basal insulin

Insulin regimens	Discontinuation of basal insulin at v2 (%)	Discontinuation of basal insulin at v3 (%)
Total	3016 (100.0)	4616 (100.0)
Only bolus	112 (3.7)	177 (3.8)
Shift to premixed	881 (29.2)	1197 (25.9)
Stop all insulin	2023 (67.1)	3242 (70.2)

Note

v2: 3 mo; v3: 6 mo.

Compared with BI discontinuers, those who persisted with BI treatment during the 6‐month follow‐up period had lower FPG (7.6 vs 8.7 mmol/L, adjusted *P* < .0001) and HbA1c levels (7.4% vs 7.6%, adjusted *P* < .0001) at v3, fewer general hypoglycaemic events in the past month (0.17 vs 0.25, adjusted *P* = .0002), lower proportion of general hypoglycaemic events in the past month (7.7% vs 9.7%, adjusted *P* < .0001), less weight gain at v3 (0.06 kg vs 0.22 kg, adjusted *P* = .0122) and lower proportion of weight gain for more than 2 kg (12.1% vs 16.0%, adjusted *P* < .0001). BI continuers showed greater reductions of FPG (−3.9 vs −3.5 mmol/L, adjusted *P* < .0001) and HbA1c (−2.24 vs −2.18, adjusted *P* = .0036) at v3 compared to v1, and higher control rate of FPG (<7.0 mmol/L) (46.4% vs 28.8%, adjusted *P* < .0001) and HbA1c (<7%) (42.3% vs 36.5%, adjusted *P* < .0001) at v3 than discontinuers (Table 3).

**Table 3:** End‐point outcomes of patients by basal insulin persistence at 6 mo

End‐point outcomes	Discontinuation of BI	Continuation of BI	*P*‐value	Adjust *P*‐value^a^
FPG (mmol/L), mean (SD)	8.7 (3.0)	7.6 (2.3)	<.0001	<.0001
Change of FPG (mmol/L), mean (SD)	−3.5 (4.8)	−3.9 (4.0)	.0003	<.0001
FPG control rate (<7.0 mmol/L), (%)	28.8	46.4	<.0001	<.0001
HbA1c (%), mean (SD)	7.6 (1.6)	7.4 (1.3)	<.0001	<.0001
Change of HbA1c (%), mean (SD)	−2.18 (2.22)	−2.24 (2.23)	.0710	.0036
HbA1c control rate (<7%), (%)	36.5	42.3	<.0001	<.0001
General hypoglycaemia in the past month (times/mo), mean (SD)	0.25 (1.0)	0.17 (1.1)	<.0001	.0002
General hypoglycaemia in the past month, (%)	9.7	7.7	<.0001	<.0001
Weight gain (kg), mean (SD)	0.22 (3.3)	0.06 (2.8)	.0069	.0122
Weight gain >2 kg, (%)	16.0	12.1	<.0001	<.0001

^a^ Adjusted for regions, hospital level (secondary or tertiary), patient recruitment setting (inpatient or outpatient clinic), age, education level, location (rural or urban), BMI at v3, duration of diabetes, SMBG frequency at v3 and number of OAD types at v3.

### 3.3 Factors associated with BI treatment discontinuation

**Figure 1:**
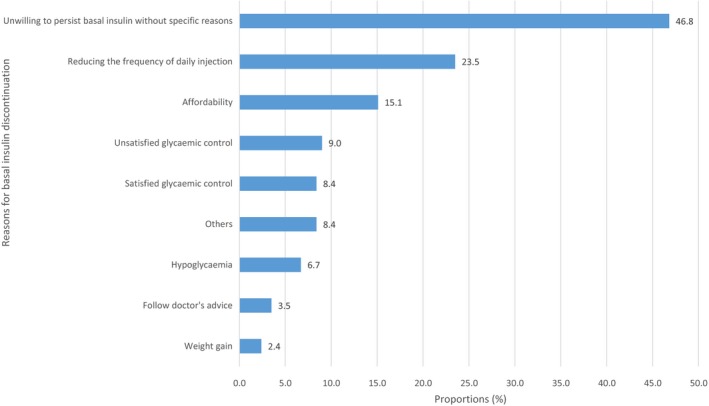
Proportion of reasons for basal insulin discontinuation by multiple choices question asking patients the reasons for discontinuation

Of those who discontinued BI therapy, the most common reason reported for BI discontinuation was that they were unwilling to persist BI without specific reasons (46.8%), followed by reducing the frequency of injection (23.5%), affordability of BI cost (15.1%), unsatisfied (9%) or satisfied glycaemic control (8.4%), hypoglycaemia (6.7%), following doctor's advice (3.5%), weight gain (2.4%) and other reasons (8.4%) (Figure 1).

**Figure 2:**
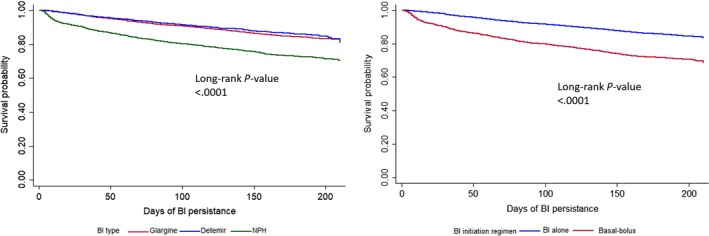
Kaplan‐Meier curves for the 6‐mo basal insulin treatment persistence after initiation by basal insulin type and insulin regimen

Multivariate Cox regression analysis showed that higher BI discontinuation was associated with the following factors: tertiary hospital, inpatient, younger age, lower education level, higher out‐of‐pocket ratio, higher BMI level, longer diabetes duration, lower SMBG frequency, initiating BI type of NPH and initiating with basal‐bolus at baseline (Table 4). Figure 2 also shows that initiating with NPH and basal‐bolus insulin were associated with significantly earlier BI discontinuation, compared with initiating glargine or detemir, and BI only, respectively.

**Table 4:** Cox regression analyses of discontinuation of basal insulin

Baseline variables	Unadjusted hazard ratio (95% CI)	*P*‐value	Adjusted hazard ratio (95% CI)^a^	*P*‐value
Tertiary hospital vs Secondary hospital	1.136 (1.072‐1.204)	<.0001	1.268 (1.192‐1.349)	<.0001
Inpatient vs outpatient clinic	1.474 (1.388‐1.565)	<.0001	1.317 (1.229‐1.411)	<.0001
Age (y)	0.995 (0.992‐0.998)	.0004	0.996 (0.993‐1.000)	.0243
Education (vs Primary school or lower)				
Junior high school	0.822 (0.764‐0.884)	<.0001	0.843 (0.780‐0.912)	<.0001
Senior high school	0.735 (0.679‐0.796)	<.0001	0.801 (0.732‐0.877)	<.0001
Junior college or higher	0.643 (0.586‐0.705)	<.0001	0.713 (0.639‐0.796)	<.0001
Urban residence vs rural residence	1.227 (1.155‐1.303)	<.0001	0.925 (0.854‐1.002)	.0556
Out‐of‐pocket ratio (%)	1.005 (1.004‐1.006）	<.0001	1.004 (1.002‐1.005）	<.0001
BMI group (kg/m^2^), (vs <24)				
24‐27	0.969 (0.910‐1.033)	.3331	1.062 (0.996‐1.134)	.0671
≥28	1.099 (1.011‐1.195)	.0263	1.154 (1.058‐1.258)	.0012
Diabetes duration (y)	0.973 (0.967‐0.979)	<.0001	0.972 (0.966‐0.978)	<.0001
Times of SMBG per month before initiating BI (vs 0)				
1‐5	0.868 (0.814‐0.927)	<.0001	0.975 (0.911‐1.042)	.4506
≥6	0.726 (0.671‐0.786)	<.0001	0.910 (0.839‐0.988)	.0243
Number of OAD types concomitant with BI (vs 0)				
1	0.571 (0.532‐0.614)	<.0001	0.847 (0.730‐0.982)	.0275
≥2	0.547 (0.508‐0.589)	<.0001	0.767 (0.586‐1.003)	.0526
BI type (vs Glargine)				
Detemir	0.958 (0.873‐1.052)	.3679	0.918 (0.835‐1.009)	.0763
NPH	1.742 (1.627‐1.864)	<.0001	1.558 (1.443‐1.681)	<.0001
Basal‐bolus insulin used at baseline (vs BI only)	2.319 (2.185‐2.461)	<.0001	1.857 (1.706‐2.020)	<.0001

^a^ Adjusted for all listed covariates.

## 4 DISCUSSION

This real‐world study shows that 25.8% discontinued BI therapy within 6 months after initiation, with nearly two‐thirds doing so within the first 3 months. Among those nonpersistent patients, 70.2% stopped all insulin therapy, 25.9% switched to premixed insulin and 3.8% switched to bolus only. Three most common reasons for BI discontinuation were unwilling to persist BI without specific reasons (46.8%), reducing the frequency of daily injection (23.5%) and medical affordability (15.1%). Factors significantly associated with BI discontinuation were hospital level, patient recruitment setting, age, education level, out‐of‐pocket ratio, BMI, diabetes duration, SMBG, BI types and insulin regimen. Compared with discontinuers, those who continued BI therapy had higher FPG and HbA1c control rate, less general hypoglycaemia and weight gain.

The 6‐month discontinuation rate of BI therapy after BI initiation in this study was higher than or similar to the 1‐year rates reported by some studies. A study based on administrative claims from US‐based companies found that 18% of patients discontinued BI therapy in the year after initiation^13^; and a retrospective cohort study based on the French national health insurance database treatment reported a discontinuation rate of 25% during the first year.^15^ Those might imply that our study revealed a higher proportion of patients discontinued BI therapy in 6 months in comparison. However, it is similar to a study using questionnaire‐based phone interview survey, in which 24% were reported to discontinue insulin therapy during 6 months after initiation.^17^ The difference in discontinuation estimates may also be attributed to the different definitions of discontinuation and data resources. Perez‐Nieves et al^13^ classified patients as continuers (no gap), interrupters (≥1 prescription after gap) and discontinuers (no prescription after gap) based on whether patients had ≥30 day gaps in BI use in the first year post‐index. Roussel et al^15^ defined discontinuation as the absence of reimbursement for insulin over 6 months or 1 year after the initiation date using insurance claims. By comparison, we defined discontinuation as discontinuing the BI reported by physicians at 3‐month visit or 6‐month visit, which included switching to other insulin type such as bolus only or premixed insulin, or stopping all insulin therapy. As we did not separate interrupters from discontinuers, those who discontinued BI at 3‐month visit but resumed BI at 6‐month visit were also classified as discontinuation. Though, only 1.4% 3‐month discontinuers were reported to resume BI therapy at 6 months in our study. In addition, as patients were followed up for 6 months, we could not examine whether their insulin discontinuation reflected a temporary interruption or a complete cessation.

In this study, almost half of patients who discontinued BI therapy reported being unwilling to persist BI without specific reasons as a main reason for BI discontinuation. Those patients might have difficulties in integrating insulin use into their daily lifestyle due to regimen complexity. Therefore, it is critical to improve convenience and flexibility of insulin use such as fewer injections^18^, ^19^ and the use of insulin pen delivery devices.^19^, ^20^ Moreover, 23.5% of discontinuers reported reducing the frequency for injection as a reason for BI discontinuation, and about a quarter of them switched to premixed insulin with once‐ or twice‐daily injections instead of basal‐bolus insulin with four times daily injections (1 BI injection + 3 bolus injections). Affordability of insulin therapy was the third most common reason for BI discontinuation, accounting for 15.1%. In line with our finding, a previous multinational survey also showed 9.8% and 17.2% of patients reported cost of therapy as a reason for interruption and discontinuation, respectively.^21^ And multivariate Cox regression analysis also indicated that ≥80% out‐of‐pocket ratio was associated with higher likelihood of discontinuation compared with <20% out‐of‐pocket ratio. Although cost‐related factors play a role in causing treatment discontinuation, patients may be reluctant to report difficulty paying for their medication.^22^, ^23^ Physicians should raise discussion with their patients about cost‐related problems with treatment discontinuation and make it clear that cost is an important issue, and should not cause embarrassment. They should also provide their patients with information on sources of inexpensive medications and financial assistance programmes to appropriately adjust treatment to minimize costs.

Although glucose control effect of insulin therapy in patients with T2DM has been widely acknowledged, uncontrolled hyperglycaemia was reported by patients as one of the reasons for interrupting/discontinuing insulin therapy after initiation.^24^ In this study, 9% of discontinuers self‐reported unsatisfied glycaemic control as the reason for their discontinuation. Another study reported 11.4% and 10.1% interrupted and discontinued insulin therapy due to insufficient glycaemic control, respectively.^21^ Those patients might perceive insulin therapy as lack of short‐term clinical efficacy, therefore, be unwilling to persist in it. In these instances, it is crucial for physicians to determine the reasons of uncontrolled hyperglycaemia, to discuss with patients about the benefits of insulin therapy and the consequences of uncontrolled hyperglycaemia. And to decide which step should be taken: insulin dose titration or treatment regimen switch. On the other hand, our study showed that 8.4% of patients discontinued BI therapy due to satisfied glycaemic control. For those patients, physicians might use short‐term BI therapy to reduce glycaemia to a level that OADs might be able to maintain. It could be a temporary cessation if glucose cannot be controlled by OADs after discontinuation, and resumption of insulin therapy might be needed.

Compared with previous studies,^21^, ^25^ we found that much lower proportions of discontinuers reported hypoglycaemia (6.7%) or weight gain (2.4%) as the reasons for discontinuation. In this study, general hypoglycaemia happened in 9.7% and 7.7% of discontinuers and continuers in the past month (weight gain >2 kg: 16.0%, 12.1%), respectively, whereas the proportion of severe hypoglycaemia after BI initiation was very low (0.6%). These results indicated that part of patients who experienced hypoglycaemia or weight gain might be tolerant of those side effects and continued BI therapy. Moreover, hypoglycaemia might have a higher impact on BI discontinuation than weight gain. However, in Cox regression analysis, patients with BMI ≥28 kg/m^2^ were more likely to discontinue BI therapy compared with those with BMI <24kg/m^2^, which implied that patients with higher BMI were more concerned with weight gain caused by insulin therapy and therefore more likely to discontinue BI therapy.

Among BI discontinuers, 1522 (36.5%) in total had good glycaemic control (HbA1c <7%) after 6 months, indicating physicians may prescribe BI prematurely as they had not adjusted dose of OADs appropriately before insulin treatment. In 8178 patients taking only one OAD before insulin treatment, 2225 (27.2%) stopped BI after 6 months with 842 patients reporting good glycaemic control (HbA1c <7%). In addition, patients with two OADs may also start insulin prematurely. We found that inpatients and patients in tertiary hospitals were more likely to discontinue BI therapy compared with outpatients and those in secondary hospitals, respectively. It might be due to a higher level of hyperglycaemia among patients in tertiary hospitals and inpatients, and physicians tend to initiate intensive insulin therapy consisting of BI and bolus insulin for a short term until glycaemia was controlled, and then returned to only OADs. As another prescribers' factor, physicians in rural and secondary hospitals were more likely to stop metformin after insulin initiation (rural vs urban: 32.0% vs 24.7%; secondary vs tertiary: 30.2% vs 24.9%), which indicating prescribers in rural areas and lower level hospitals had more deviation from the guideline. We need to explore the detailed clinical features, actual reasons and related factors for the premature use of BI therapy and metformin discontinuation in our future research.

We also found that patients initiating basal‐bolus were more likely to discontinue BI therapy than those who initiated only BI. Given that most of those who initiated basal‐bolus adopted an insulin regimen with one injection of BI and three injections of bolus insulin, and about a quarter of discontinuers reported reducing the frequency of injection as the reason for discontinuation, patients initiating basal‐bolus were more likely to switch from basal‐bolus to premixed insulin or even stopping all insulin. Moreover, higher injection frequencies of NPH compared with glargine insulin may also contribute to a higher likelihood of BI discontinuation of NPH users than glargine users.^26^ And less hypoglycaemia caused by glargine, especially during the night, might also partly explain those findings.^27^, ^28^

Patients with older age and longer diabetes duration were found to be associated with lower likelihood of BI discontinuation, consistent with some previous studies.^8^, ^27^, ^29^ We also found patients with higher education level were associated with lower odds of discontinuation, compared with primary or lower education. Given patients with lower education might have more difficulties in continuing BI therapy, more efforts should be taken by physicians at treatment initiation and afterwards, such as emphasizing the benefits of treatment persistency and the adverse outcomes of non‐persistency at initiation, figuring out exact reasons for discontinuation and help patients resume treatment after discontinuation if appropriate. Moreover, patients with ≥6 times of SMBG per month before initiating BI were associated with lower odds of discontinuation compared with those without SMBG. Patients with a lower frequency of SMBG might not pay attention to their glycaemic levels and have difficulties in managing their diabetes. Therefore, physicians should give more supports of SMBG to those who have low frequency of SMBG before and after they initiate BI therapy.

This study also showed advantages of continuing BI therapy with respect to the effect on FPG and HbA1c control rate, compared with discontinuers, after 6 months of BI initiation. And among continuers, general hypoglycaemia and weight gain were also lower. It is more likely that patients continued BI therapy because of lower incidence of hypoglycaemia and weight gain.

Some limitations of the present study should be mentioned. First, patients were classified as discontinuers based on whether they stopped BI therapy at 3‐ or 6‐month visit, regardless of their resumption of BI therapy after that discontinuation. However, our further analysis showed that only about 1% of patients who stopped BI therapy at 3‐month visit were reported to start BI therapy again at 6‐month visit. Second, this study did not collect information on patients' adherence of BI therapy, so we could not estimate to what extent the patients follow the instructions of BI therapy and its impact on treatment effectiveness and safety. Finally, the length of follow‐up was only 6 months, which may not be long enough to detect the full benefits of treatment persistence.

## 5 CONCLUSION

In conclusion, among patients with T2DM who initiated BI therapy due to uncontrolled hyperglycaemia by OADs, a quarter discontinued BI therapy within 6 months. The most common reason for discontinuation reported by patients was unwilling to persist BI without specific reasons. Tertiary hospital, inpatient, younger age, lower education level, higher out‐of‐pocket ratio, higher BMI level, longer diabetes duration, lower SMBG frequency, initiating with NPH and basal‐bolus at baseline were associated with higher BI discontinuation.

## CONFLICT OF INTEREST

L.J. reported receiving consulting and lecture fees from Eli Lilly, Bristol‐Myers Squibb, Novartis, Novo Nordisk, Merck, Bayer, Takeda, Sanofi, Roche and Boehringer Ingelheim, and research grants from Roche and Sanofi. The other authors declare that they have no conflicts of interest.

## AUTHOR CONTRIBUTIONS

L.J. and P.Z. contributed to the conception and design of the study. X.L. contributed to the data analysis. P.Z., H.Z., M.C. and D.W. contributed to the drafting of the manuscript. All authors contributed to the acquisition and/or interpretation of the data. All authors critically read the manuscript, suggested revisions and approved the final version of the manuscript.

## ETHICAL APPROVAL

The ORBIT study protocol was approved by the Institutional Review Board (IRB) of Peking University and, when necessary, by local IRBs. Written informed consents were obtained from all patients before the start of study.

## Data Availability

The data sets used and/or analysed during the current study are available from the corresponding author on reasonable request.
